# Founder events and subsequent genetic bottlenecks underlie karyotype evolution in the Ibero-North African endemic *Carex helodes*

**DOI:** 10.1093/aob/mcad087

**Published:** 2023-07-03

**Authors:** Marcial Escudero, Juan Miguel Arroyo, Santiago Sánchez-Ramírez, Pedro Jordano

**Affiliations:** Department of Plant Biology and Ecology, University of Seville, 41012 Seville, Spain; Department of Integrative Ecology, Doñana Biological Station, CSIC, 41092 Seville, Spain; Department of Integrative Ecology, Doñana Biological Station, CSIC, 41092 Seville, Spain; Department of Ecology and Evolutionary Biology, University of Toronto, M5S 3B2 Toronto, Ontario, Canada; Department of Plant Biology and Ecology, University of Seville, 41012 Seville, Spain; Department of Integrative Ecology, Doñana Biological Station, CSIC, 41092 Seville, Spain

**Keywords:** Chromosome, Cyperaceae, dysploidy, genetic drift, holocentric chromosomes, inbreeding, selfing

## Abstract

**Background and Aims:**

Despite chromosomal evolution being one of the major drivers of diversification in plants, we do not yet have a clear view of how new chromosome rearrangements become fixed within populations, which is a crucial step forward for understanding chromosomal speciation.

**Methods:**

In this study, we test the role of genetic drift in the establishment of new chromosomal variants in the context of hybrid dysfunction models of chromosomal speciation. We genotyped 178 individuals from seven populations (plus 25 seeds from one population) across the geographical range of *Carex helodes* (Cyperaceae). We also characterized karyotype geographical patterns of the species across its distribution range. For one of the populations, we performed a detailed study of the fine-scale, local spatial distribution of its individuals and their genotypes and karyotypes.

**Key Results:**

Synergistically, phylogeographical and karyotypic evidence revealed two main genetic groups: southwestern Iberian Peninsula vs. northwestern African populations; and within Europe our results suggest a west-to-east expansion with signals of genetic bottlenecks. Additionally, we inferred a pattern of descending dysploidy, plausibly as a result of a west-to-east process of post-glacial colonization in Europe.

**Conclusions:**

Our results give experimental support to the role of geographical isolation, drift and inbreeding in the establishment of new karyotypes, which is key in the speciation models of hybrid dysfunction.

## INTRODUCTION

Chromosomal evolution is one of the major drivers of diversification in eukaryotes (Coghlan *et al.*, 2005) as it has been recognized to affect all branches of the angiosperm tree of life (Carta *et al.*, 2020). In fact, it is now well known that all angiosperms have undergone several cycles of polyploidization and subsequent post-polyploid diploidizations (Wendel, 2015; Escudero and Wendel, 2020). The importance of polyploidization in angiosperms has long been acknowledged by many botanists (Soltis *et al.*, 2015). In fact, it is thought that the origin of angiosperms was preceded by an event of whole genome duplication (Jiao *et al.*, 2011). More recently, the importance of dysploid events has also been recognized in the evolution of angiosperms (Escudero *et al*., 2014), especially during the process of post-polyploid diploidization (Wendel, 2015; Escudero and Wendel, 2020).

There are two main kinds of models of chromosomal speciation, suppression of recombination of locally adapted supergenes and hybrid dysfunction models (Coyne and Orr, 2004). The model involving suppression of recombination is a speciation model compatible with ongoing gene flow between chromosomal variants, where the total absence of reduced fertility between chromosomal hybrids is observed (Butlin, 2005). The basis of this model is the existence of at least one region in the genome where there is no recombination during meiosis mainly due to non-homology across a given genomic region (Faria and Navarro, 2010). Typically, the non-homology is generated by the existence of a chromosome rearrangement, such as an inversion, giving rise to two chromosomal variants (Rieseberg, 2001). The non-recombinant region is generally recognized as a supergene that is locally adapted (Lowry and Willis, 2010). The other main kind of chromosomal model of speciation involves hybrid dysfunction, leading to reduced fitness in individuals carrying two chromosomal variants (Coyne and Orr, 2004). This model has been criticized by the fact that highly underdominant chromosome mutations are difficult to establish in a population mostly composed of other chromosomal variants (Coyne and Orr, 2004). In contrast, a weakly underdominant chromosomal mutation has a higher probability to be fixed in a new population but will not cause differentiation and speciation as a result of hybrid dysfunction (Butlin, 2005). Rieseberg (2001) summarized the most important known models of hybrid dysfunction in chromosomal speciation (some of them untested experimentally). In every case the mechanistic basis leading to an increase in the probability of establishment of chromosomal variants can involve (1) genetic drift, (2) inbreeding, (3) selection in favour of individuals that are homozygous for the new variant, and (4) meiotic drive (White, 1978).

In eukaryotes, there are two main kinds of chromosomes based on the structure of the kinetochore activity: monocentric and holocentric chromosomes. Holocentric chromosomes have kinetochore activity along the whole chromosome instead of having such activity concentrated in a single point in the chromosome known as the centromere (Hipp *et al*., 2013), and ~15–20 % of the species in the eukaryotes may have this kind of chromosome including animals, plants and rhizaria (Márquez-Corro *et al.*, 2019). In the animal kingdom, 12 different important lineages have this type of chromosome (Márquez-Corro *et al.*, 2018). In the plant kingdom, holocentric chromosomes appear in three eudicot angiosperms, namely the genera *Myristica* (i.e. *M. fragrans*, Myristicaceae, order Magnoliales), *Drosera* (Droseraceae, order Caryophyllales) and *Cuscuta* (Convolvulaceae, order Solanales), and in two monocotyledonous lineages, namely Melanthiaceae (order Liliales) and the Cyperaceae plus Juncaceae clade (order Poales) (reviewed in Márquez-Corro *et al.*, 2018).

Holocentricity has several implications for the evolution of chromosomes. In monocentric chromosomes, after chromosome fission, chromosome fragments without centromeres are unable to segregate normally. Therefore, chromosome fissions are expected to result in a loss of genetic material during meiosis and inviable gametes. However, in holocentric chromosomes, diffuse centromeres allow chromosome fragments to segregate normally during meiosis (Faulkner, 1972; Luceño and Castroviejo, 1991). Holocentricity may thus promote chromosome number variation via fission and fusion, as these mutations are expected to be neutral or nearly so in holocentric organisms (Hipp *et al*., 2013). Two holocentric groups show extraordinary chromosome number variation: (1) the insect order Lepidoptera, i.e. the families Lycaenidae (*Agrodiaetus* butterflies, 2*n* = 20–268, Lukanov *et al.*, 2005) or Nymphalidae (tribe Ithomiini, 2*n* = 10–240, Joron *et al.*, 2011), and (2) sedges, the angiosperm family Cyperaceae (2*n* = 4–226, Márquez-Corro *et al.*, 2019, and especially the genus *Carex*, 2*n* = 12–124, Escudero *et al.*, 2012). Thus, holocentric organisms have been thought as highly suitable models to study chromosomal speciation (Lucek *et al.*, 2022).

The specific goals of this study were to: (1) disentangle the genetic structure of *Carex helodes* among and within populations, (2) to understand the geographical structure of chromosomal variants, and (3) to infer the patterns of how chromosomal variants become established by joining two sources of information: genetic and karyotypic. Overall, we aim to test the hypothesis that the establishment of chromosomal variants may be produced during the foundational process of new populations when the species expands its geographical range (through the combined action of genetic drift, inbreeding and geographical isolation). We also hypothesize that holocentricity may help in the process of chromosomal speciation by facilitating the establishment of new karyotypes (Lucek *et al.*, 2022).

To test the hypothesis that chromosomal variants may establish during the process of expansion and foundation of new populations, we need to know in detail both the phylogeographical history of the species and the distribution of chromosomal variants. In this study we combine both sources of information and two different geographical scales for the purpose of understanding the chromosomal evolutionary history of the species and its possible implications for differentiation and speciation.

## MATERIAL AND METHODS

### Study species

*Carex helodes* Link from *Carex* sect. *Spirostachyae* (Drejer) L.H. Bailey (Global Carex Group, 2015, 2016, 2021; Villaverde *et al.*, 2020) in the family Cyperaceae (Larridon *et al.*, 2021) is a diploid, wind‐pollinated, perennial herb (typical generation time is 2 years although in good growing conditions a generation time of 1 year has been observed). This species is well characterized by its caespitose habit (without creeping rhizomes), rough upper leaf surface, high number of male spikes (1–4[–7]) and androgynous spikes. The species grows in temporarily inundated acidic soils in open habitats within corn oak woodlands. This species is endemic to southern Portugal, southwestern Spain and northern Morocco (in the last two countries it is rare). The species is listed in the IUCN red list as Near Threatened (Rankou, 2018). Chromosome number ranges from 2*n* = 70 to 2*n* = 75 (Escudero *et al.*, 2008, 2010). *Carex helodes* has been intensely studied in recent years. Escudero *et al.* (2008), using chromosome counts and AFLP (amplified fragment length polymorphism) genotypes from nine populations across the species’ distribution, demonstrated that (1) the species is monophyletic, (2) major genetic differences occur between European and African populations with low genetic differentiation within the Iberian Peninsula and within Africa, and (3) Portuguese populations are characterized by a diploid chromosome number 2*n* = 72 while Moroccan populations by a diploid chromosome number 2*n* = 74. Escudero *et al.* (2010) found that Spanish populations have mixed chromosome numbers, 2*n* = 72 and 2*n* = 70. Arroyo *et al.* (2016), using 454 pyrosequencing of microsatellite-enriched DNA, designed 91 polymorphic loci for *C. helodes* (and established a set of the 34 most variable microsatellites).

### Sampling

**Fig. 1.**
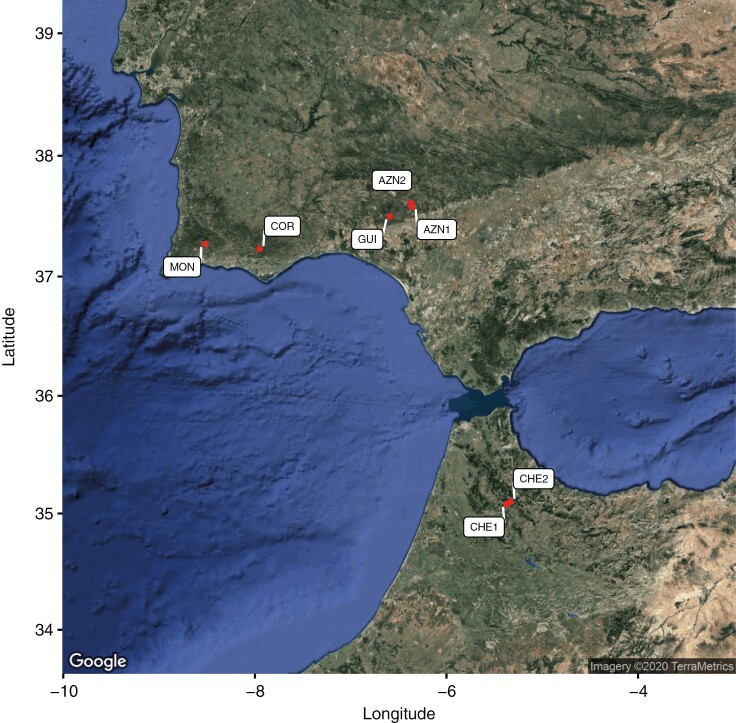
Satellite image of southern Spain and Portugal and northern Morocco with the locations of the seven studied populations (CHE1, CHE2, COR, MOR, GUI, AZN1 and AZN2; see Table 1).

**Fig. 2.**
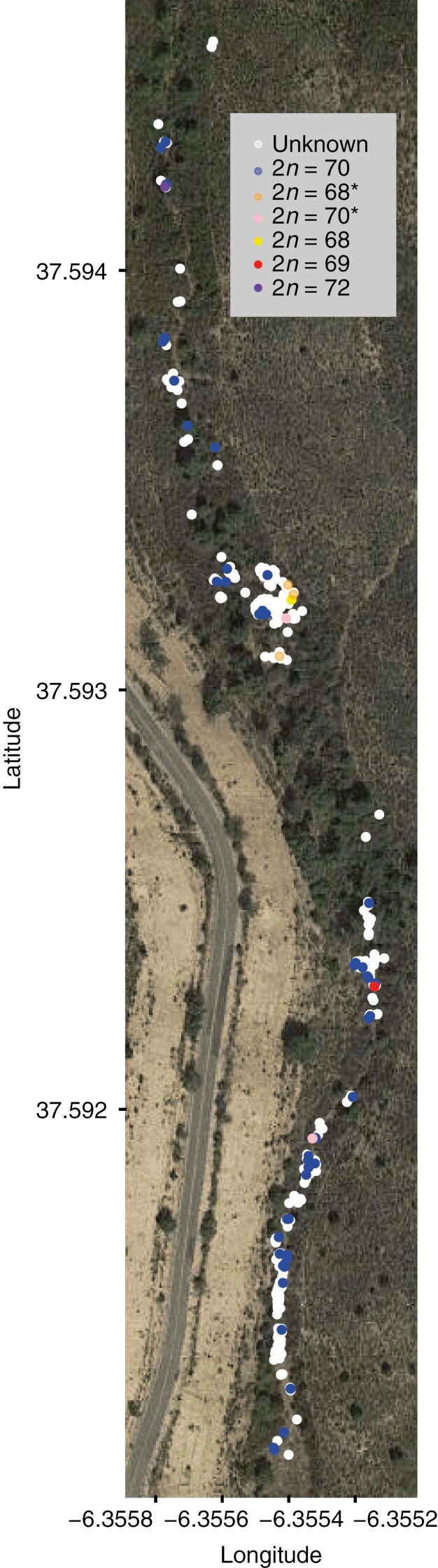
Location of all individuals during spring 2014 in the population AZN1. The population is contextualized using a satellite image. Individuals with unknown chromosome number are indicated in white. The most common regular chromosome number 2*n* = 70 is shown in blue. The regular chromosome numbers 2*n* = 68 and 2*n* = 72 are shown in yellow and purple, respectively. The irregular chromosome numbers of configurations 2*n* = 68*, 2*n* = 69 and 2*n* = 70* are shown in orange, red and pink, respectively. Latitude and longitude are indicated in the vertical and horizontal axis, respectively. Note: we use an asterisk to indicate the presence of irregularities in paired diploid chromosome numbers.

We collected 178 individuals (plus 25 seeds) from seven populations across the range of distribution of *C. helodes* (Fig. 1). For the population AZN1 (Table 1), we georeferenced all individuals during the spring 2014 with a very sensitive GPS device (model Leica 1200 rover) that is able to provide geographical positions with a precision of millimetres (a total of 399 individual were georeferenced, Fig. 2, Supplementary Data Fig. S1). We collected leaf materials of 47 individuals for simple sequence repeat (SSR) genotyping study and young flowers of 56 individuals for cytogenetic studies across the whole population (the intention was to obtain the karyotype of the same individuals collected for genotyping but because of the difficulty of determining the karyotype of specifically selected individuals in the populations this was not always possible). We also collected 25 seeds from 25 different individuals in this population representing the whole range of the population (Table 1). Additionally, leaf material of 131 individuals from six populations (from 15 to 26 individuals per population) was collected (Table 1) from southwestern Spain, southern Portugal and northern Morocco, covering most of the distribution of the species. From previous studies (Escudero *et al.*, 2008, 2010) we also gathered cytogenetic information from 23 individuals for five of these additional six sampled populations (—two to seven individuals sampled per population, Table 1).

**Table 1.** Sampling information. Population code, country, locality and geographical latitude and longitude. Sampling details for cytogenetic and genotyping studies are also indicated. The cytogenetic results are also provided.

Code	Country	Locality	Longitude/latitude	*N* Cyt.	2*n*	*N* SSR
CHE1	Morocco	Tanger-Tetuan: Chauen – Ksar el Kebir	−5.37/35.08	5	74	15
CHE2	Morocco	Tanger-Tetuan: Chauen – Ksar el Kebir	−5.33/35.10	2	74	16
COR	Portugal	Algarve: Corthela	−7.95/37.23	7	72	25
MON	Portugal	Algarve: Barraçao – Caldas de Monchique	−8.52/37.27	5	4 × 72, 1 × 75*	26
GUI	Spain	Huelva: El Guijo	−6.59/37.50	4	2 × 70, 2 × 72	22
AZN1	Spain	Sevilla: Aznalcollar – El Álamo	−6.36/37.58	56	1 × 68, 3 × 68* (33^II^ + 2^I^ or 31^II^ + 2^III^), 1 × 69* (33^II^ + 1^III^), 48 × 70, 2 × 70* (34^II^ + 2^I^), 1 × 72	47 (25 F_1_)
AZN2	Spain	Sevilla: Aznalcollar—El Álamo	−6.37/37.61	–	–	27

### Cytogenetic analyses

Meiotic plates for the 56 individuals sampled from AZN1 (Table 1) were prepared following Luceño (1988). The number of homologous chromosomes (bivalents) was observed during pairing in metaphase of meiosis I. We also studied the pairing irregularities (trivalents, tetravalents, etc.) during the metaphase of meiosis I. Univalents are denoted with ‘I’, bivalents with ‘II’, trivalents with ‘III’, etc.

### SSR genotyping and analyses

Genotyping for the 203 individuals sampled (178 plants and 25 seeds) from the seven populations (Table 1) was performed using the 34 microsatellites previously selected because of their variability by Arroyo *et al.* (2016) among the 91 polymorphic microsatellites that were designed. The laboratory procedure for DNA extraction, microsatellite amplification through PCR, sequencing and scoring was identical to the one followed by Arroyo *et al.* (2016).

With the dataset of the 178 sampled plants and 34 loci, we obtained different estimates of genetic diversity indices of populations and loci using the functions poppr and locus.table implemented in the R poppr package (Kamvar *et al.*, 2014, 2015). We inferred the neighbour-joining (NJ) tree and a minimum spanning network using the functions bruvo.boot and bruvo.msn as implemented in the R package poppr using Bruvo’s distance, which uses the length of the nucleotide repeats for each microsatellite locus (Bruvo *et al*., 2004). For comparison, we also inferred the NJ tree using the function aboot as implemented in the R package poppr (Kamvar *et al.*, 2014, 2015) using Nei’s distance (Nei, 1972, 1978). We estimated NJ branch support using 1000 non-parametric bootstrap replicates. NJ trees were rooted based on BEASTvntr inference.

To identify genetic structure within our data set of 178 individuals and 34 loci, we used a Bayesian approach under an explicit population genetic model to find clusters of individuals under Hardy–Weinberg equilibrium and random mating and allowing admixture of populations and individuals. STRUCTURE v.2.3.2 (Pritchard *et al.*, 2000) uses a Markov chain Monte Carlo (MCMC) method to recover a posterior probability (PP) distribution of population partitions and population genetic parameters. In STRUCTURE analyses, we utilized the admixture, correlated allele frequencies model. STRUCTURE simulations were run from *K* = 1 to 10 populations, with ten replicates per run of 500 000 iterations with a burn-in of 100 000 iterations. The best-fit value of *K* was estimated using the online server STRUCTURE HARVESTER (Earl and VonHoldt, 2012), taking into account the *K* selection concerns raised by Janes *et al.* (2017). The admixture graphic was generated using STRUCTURE PLOT (Ramasamy *et al.*, 2014).

A chronogram with absolute times was estimated using 33 SSRs (one was excluded because it had missing data) and 176 individuals (two individuals were also excluded for having missing data) in BEASTvntr implemented in BEAST2 (Drummond *et al.*, 2012; Bouckaert *et al.*, 2014). Clock, tree and site models were linked for the 33 SSRs following recommendations in the BEASTvntr manual (https://github.com/arjun-1/BEASTvntr). We used a constant coalescent model as tree prior and branching times modelled under a strict clock prior. For the site model, we used the *Sainudiin Computed Frequencies Vanilla Gamma* (Wu and Drummond, 2011), which is a modification of the original model *Sainudiin Vanilla* (Sainudiin *et al.* 2004). Analyses were conducted using two independent MCMC runs of 10 million generations each. Convergence, burn-in and effective sample sizes (ESS > 200) for each parameter were assessed in Tracer v.1.7 (Rambaut *et al.*, 2018). The trees and consensus trees were plotted using DensiTree (Bouckaert, 2010). We used a mutation rate of 1 × 10^−5^ loci per generation, which is in the range of the slowest SSR mutation rates reported for plants (Marriage *et al.*, 2009). We also conducted an analysis assuming 1 × 10^−4^ loci per generation, which is the average of mutation rates in plants. This mutation rate was chosen based on microsatellite mutation rates in two cereal crops, durum wheat and maize (Thuillet *et al.*, 2002; Vigouroux *et al.*, 2002).

## RESULTS

### Cytogenetic results

Most of the individuals (48 of 56, ~85%) in the AZN1 population displayed a regular diploid chromosome number of 2*n* = 35^II^ = 70. The remaining ~15% of the population displayed a different chromosome number or meiotic configuration. One individual displayed a regular diploid chromosome number of 2*n* = 34^II^ = 68 and another individual a regular diploid 2*n* = 36^II^ = 72. Six individuals displayed irregular chromosome numbers: three individuals displayed 2*n* = 68 with irregularities (2*n* = 33^II^ + 2^I^ or 31^II^ + 2^III^), one individual 2*n* = 69 (2*n* = 33^II^ + 1^III^) and two individuals 2*n* = 70 (2*n* = 34^II^ + 2^I^) (see Table 1).

The geographical locations of all chromosome counted individuals in AZN1 in comparison with the location of all individuals in spring 2014 are shown in Fig. 2. All individuals of *C. helodes* in AZN1 were next to a creek with stationary water with the exception of a temporary wet meadow where *C. helodes* individuals were found outside of the creek. Forty-eight of 56 individuals showed a regular chromosome number of 2*n* = 70. Five of the eight individuals with a different chromosome number (one individual with 2*n* = 68 and three individuals with 2*n* = 68*) [an asterisk indicates the presence of irregularities in paired diploid chromosome numbers] or meiotic configuration (one individual with 2*n* = 70*) were clustered in the temporarily wet meadow. The other three individuals with different chromosome number (2*n* = 72 and 2*n* = 69) or meiotic configuration (2*n* = 70*) were found along the creek and surrounded by individuals with regular 2*n* = 70.

### SSR genotyping

The genotyping rate was near 100% (99.54%). Supplementary Data Table S1 displays diversity statistics for each of the 34 SSR loci used in this study. The populations from Portugal displayed the highest levels of diversity, followed by the Moroccan populations (Table 2). Both observed and expected heterozygosity were very low for each population, although the overall expected heterozygosity was much higher than the overall observed heterozygosity (Table 2). The populations from Spain displayed extremely low levels of genetic diversity (with only one or two genotypes per population and no private alleles) and both expected and observed heterozygosity were extremely low (Table 2). The 25 genotyped seeds in population AZN1 were also identical to their mothers.

**Table 2.** Population name, sampling (*N* = number of individuals), number of genotypes (MGL) and expected number of genotypes (eMGL) and its error (SE), Simpson’s index of diversity (lambda), evenness (E.5), expected (Hexp) and observed heterozygosity (Hobs), and private alleles (Pri.Al.) are shown for the seven sampled populations and the mean across populations. Note: Evenness (E.5) was not calculated for AZN1 because of the total absence of variability.

Pop	*N*	MLG	eMLG	SE	Lambda	E.5	Hexp	Hobs	Pri.Al.
CHE1	15	9	9.000	0.000	0.853	0.849	0.135	0.053	5
CHE2	16	9	8.688	0.464	0.836	0.789	0.123	0.101	15
MON	25	23	14.300	0.653	0.954	0.962	0.352	0.244	21
COR	26	26	15.000	0.000	0.962	1.000	0.434	0.251	45
GUI	22	2	1.682	0.466	0.087	0.468	0.048	0.088	4
AZN1	47	1	1.000	0.000	0.000	NA	0.030	0.059	0
AZN2	27	2	1.556	0.497	0.071	0.448	0.030	0.058	0
Total	178	71	8.785	1.781	0.814	0.248	0.543	0.122	

The NJ trees based on two different genetic distances (Nei and Bruvo) were largely congruent (Supplementary Data Figs S2 and S3). Both trees showed the Moroccan populations as sister groups to the remaining populations [100% bootstrap support (BS) in Nei, 54% BS in Bruvo]. The individuals from Moroccan populations CHE1 and CHE2 were mixed (Fig. S1). All the populations from the Iberian Peninsula were monophyletic with the exception of AZN1 and AZN2 that were mixed in the trees. In the NJ tree based on Nei’s distances the Portuguese MON population was sister to Spanish populations but in the NJ tree based on Bruvo’s distances the Portuguese COR population was sister to the Spanish populations. Nevertheless, these sister relationships were not supported by either of the two trees. Finally, the Spanish population GUI from Huelva was sister to the Sevillian populations (AZN1 and AZN2) in both trees, but was statistically well supported (BS 99.9%) in the tree based on Nei’s distances.

**Fig. 3.**
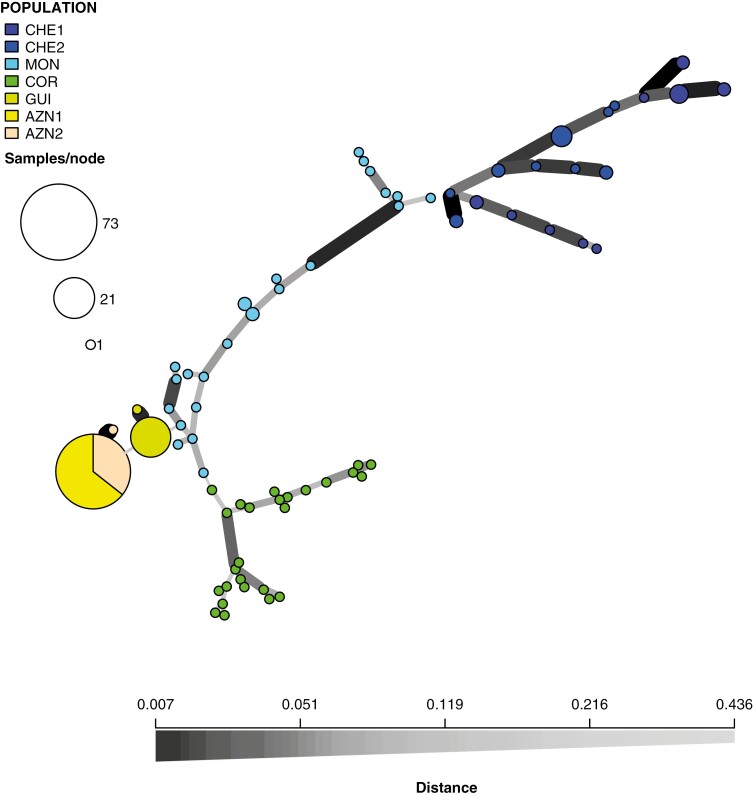
Minimum spanning network based on Bruvo’s distances, with population coding as in Table 1. The population origin of the genotypes is indicated by colours. Each circle is a different genotype and size of the circles is proportional to the number of individuals that share that genotype. The genetic distance between the genotypes is indicated by the darkness of the connections, black being the shortest genetic distance and white the largest (see scale at bottom).

The network based on Bruvo’s distances (Fig. 3) displayed similar results. There are two main groups of genotypes connected by long genetic distances: southwestern Europe vs. northwestern Africa. Within the Iberian Peninsula, the MON population was in a central position connected to the other Portuguese population and to the Spanish population GUI (with two genotypes). The populations from Seville province, AZN1 and AZN2, were connected to the GUI population from Huelva province.

**Fig. 4.**
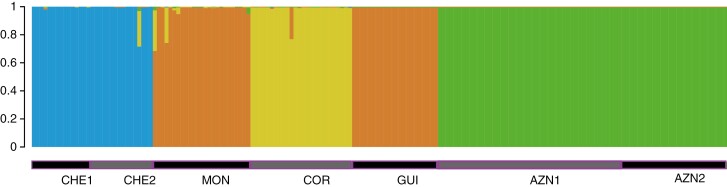
Results from STRUCTURE analyses. STRUCTURE plot representing the four inferred clusters. The populations are indicated using black and grey horizontal bars. The clusters are indicated with colours (blue for the cluster of CHE1 and CHE2, orange for the cluster of MON and GUI, yellow for the cluster of COR, and green for the cluster of AZN1 and AZN2).

The best STRUCTURE clustering was *K* = 4 followed by *K* = 2 (Supplementary Data Fig. S4), grouping populations into: (1) Moroccan (CHE1 and CHE2; *F*_ST_ = 0.8300), (2) one Portuguese population (COR; *F*_ST_ = 0.2478), (3) a Portuguese and a Spanish population (MON and GUI; *F*_ST_ = 0.5397) and (4) Spanish populations (AZN1 and AZN2; *F*_ST_ = 0.9771) (Fig. 4). The second best clustering grouped the Spanish populations with the remaining ones.

**Fig. 5.**
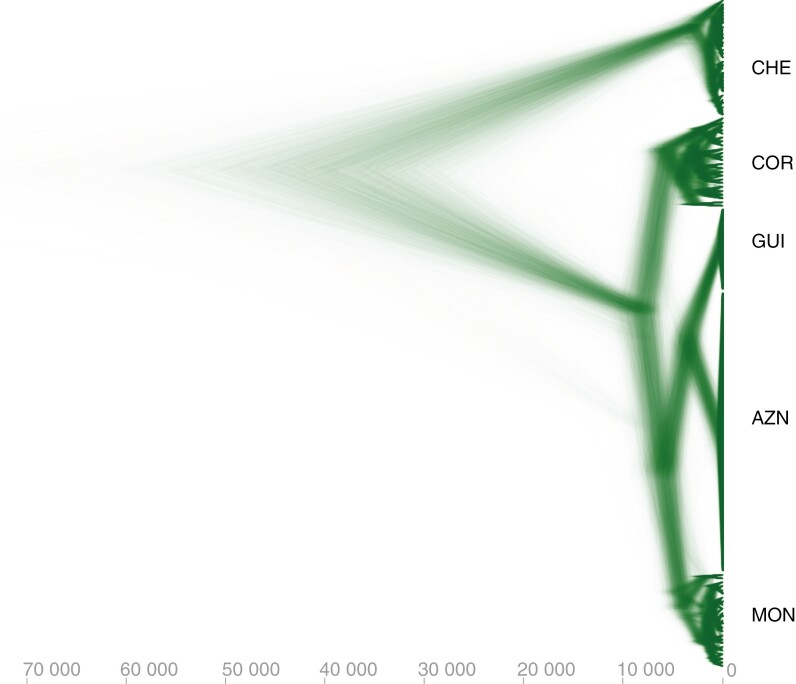
Densitree of the *Carex helodes* individuals analysed. 95% CI (confidence interval) phylogenetic trees from BEASTvntr analysis are shown in green. The *x*-axis gives the time in years. Population names are indicated.

The Bayesian trees summarized with treeannotator (Supplementary Data Fig. S5) and densitree (Fig. 5) were consistent with the NJ tree inferred with Bruvo’s distances. The relationships between major groups and populations were strongly supported. The populations from Morocco again were intermixed with and sister to the European populations (PP = 1; Fig. S5). In concordance, the European populations were monophyletic (PP = 1; Fig. S5), including the Portuguese populations COR (PP = 1; Fig. S5), which was sister to the remaining populations (PP = 0.95; Fig. S5); MOR (PP = 0.95; Fig. S5), which was sister to Spanish populations (PP = 0.98; Fig. S5); and the population from Huelva province, GUI (PP = 1; Fig. S5), sister to populations in the Sevillian province AZN1 and AZN2 (PP = 1; Fig. S5), were found to be mixed.

In terms of time of diversification we report the results based on a mutation rate of 1 × 10^−5^ loci/year (the results based on a mutation rate 1 × 10^−4^ loci/year give ages systematically 10 times younger). We report ages based on the slowest mutation rates reported for angiosperms because we inferred surprisingly young ages for divergence in *C. helodes*. We obtained an estimation for the crown node of *C. helodes* which corresponds to the split between European and African populations, 40 669 years ago [95% highest posterior density (HPD) 24 769–60 351 years]. The crown node of the African populations is at 3164 years ago (95% HPD 1778–4598 years) and the crown node of the European populations is 8218 years ago (95% HPD 6065–10 430 years). The crown nodes of the Portuguese populations COR and MOR are at 5654 years ago (95% HPD 3806–7457 years) and 4179 years ago (95% HPD 2711–5613 years), respectively. The split between Portuguese and Spanish populations was 3653 years ago (95% HPD 2239–5025 years). The crown nodes of populations GUI and AZN1–AZN2 are at 337 years ago (95% HPD 124–491 years) and 423 years ago (95% HPD 207–570 years), respectively.

## DISCUSSION

### *The phylogeographical history of* Carex helodes

*Carex helodes* is sister species to a large clade in section *Spirostachyae* and seems to be a palaeoendemic of which the stem node pre-dates the Pleistocene (Escudero *et al.*, 2010; Márquez-Corro *et al.*, 2019; Martín-Bravo *et al.*, 2019). Therefore, this species has been evolving independently from its most recent common ancestor since before the Pliocene when the climate was warmer and when temperatures started to drop moving into the Pleistocene glaciations. The present study (Figs 3 and 5) is congruent with previous phylogeographical conclusions based on AFLPs, as well as nuclear and plastid DNA regions (Escudero *et al.*, 2008). Nevertheless, this new study based on 34 SSRs allows us to go further into the genetic structure of *C. helodes* and even obtain initial estimates of population divergence times within the species (Fig. 5).

As previous studies show (Escudero *et al.*, 2008), the main genetic structure stems from the high differentiation between North African and European populations (Figs 3 and 5). The role of the Strait of Gibraltar as an effective barrier to gene flow in plants has been reported previously (Rodríguez-Sánchez *et al.*, 2008). At the species level, previous studies have found that the last closure of the Strait of Gibraltar occurred between 4.5 and 5.5 Mya, which allowed many species to migrate between Africa and Europe (Duggen *et al.*, 2003). However, the origin of most disjunct plant species in this region entails long-distance dispersal events that post-date this last closure of the Strait of Gibraltar (Escudero *et al.*, 2008; Rodríguez-Sánchez *et al.*, 2008). The current study supports this conclusion for *C. helodes*. Despite using the slowest SSR mutation rate reported for plants (see Methods), we have inferred a very recent time of differentiation between African and European populations (95% HPD 24 769–60 351 years, Fig. 5) that is compatible with the isolating effect caused by the last glacial period (*c*. 115 000 to *c*. 11 700 years ago). The higher genetic diversity among European populations compared to those in North Africa (Table 2), together with the structure shown by the DNA haplotype network (Fig. 3), suggests a long-distance dispersal and colonization from Europe to Africa and subsequent isolation and genetic differentiation (Escudero *et al.*, 2008). Long-distance dispersal events are recurrent in sedges (Martín‐Bravo *et al.*, 2019) and are probably a result of bird endozoochory (Villaverde *et al.*, 2017). In fact, bird endozoochory has been suggested as a very important dispersal syndrome in Cyperaceae (Green *et al.*, 2022). The current results based SSR genetic diversity and network structure suggest again the same conclusion of long-distance dispersal for *C. helodes*. The crown node of the European populations (95% HPD 6065–10 430 years, Fig. 5) and especially the African populations (95% HPD 1778–4598 years, Fig. 5) clearly post-date the Pleistocene. The timing of divergence between these populations probably occurred during the Holocene (*c*. 11 700 years ago to the present). The younger temporal origin of the African populations could be related to a more recent genetic differentiation or to population extinction events in more recent times. Based on AFLPs within Europe and within Africa, differentiation among populations was not found (Escudero *et al.*, 2008). In the current study, while we cannot see differentiation between the two African populations, we found a very clear genetic structure among most of the European populations which formed distinct clusters in all genetic analyses (with the exception of AZN1 and AZN2; see Figs 3–5). Our results suggest that the population MON may have been ancestral to the other European populations (see genetic network, Fig. 3; and patterns of genetic diversity, Table 2). The geographical position, in the furthest southwestern corner of Europe, could have been a suitable area serving as a refugium during glacial periods. In fact, the Algarve refugium hypothesis has been already proposed for plants in the Mediterranean Basin (Médail and Diadema, 2009). In addition, the Portuguese population COR, also in the Algarve, has a high level of genetic diversity (Table 2). The Spanish populations, with much lower levels of genetic diversity (two genotypes in GUI and two genotypes in AZN, Table 2), could be the result of recent founder events (95% HPD 2239–5025 years, Fig. 5). The crown nodes of GUI and AZN1–AZN2 are younger in comparison (95% HPD 124–491 years and 95% HPD 207–570 years, respectively, Fig. 5), which suggest a recent origin of these populations and that the colonization of Spain from Portugal involved crucial genetic bottlenecks.

### Chromosome evolution in a phylogeographical context: implications for chromosomal differentiation and speciation

Our study integrates two sources of information in a geographical context: chromosome numbers and genetic data (SSR). Having a clear picture of the phylogeography of the species allows us to track chromosomal microevolutionary processes in this species. As previously suggested (Luceño and Castroviejo, 1991; Escudero *et al.*, 2008, 2014), 2*n* = 72 is the ancestral chromosome number of *C. helodes*. This is the only regular chromosome number of the species in Portugal (2*n* = 36^II^ = 72 reported from 25 individuals from five different Portuguese populations) which is the geographical origin of the species based on molecular data (Fig. 3 and Table 2 in the current study; Escudero *et al.*, 2008). Interestingly, a higher chromosome number is found in the Moroccan populations, 2*n* = 74 (Table 1), which have remained isolated for a longer period, and is the only chromosome number reported in northern Africa (Escudero *et al.*, 2008). The species under study is likely to have suffered a descending dysploid process during the west-to-east expansion process in the Iberian Peninsula. The species shows equally frequent regular chromosome numbers of 2*n* = 72 and 2*n* = 70 in GUI, 2*n* = 70 as by far the most frequent regular number in AZN1 and a second regular but much less frequent chromosome 2*n* = 68 in this population. Interestingly, the rate of fixation of chromosomal inversions has been inferred to be very variable (4–25 inversions per 25 million generations) in a study including 32 different genera of plants (Hirabayashi and Owens, 2023). Even the fastest rates reported in their study are significantly low when compared with the rates of establishment of new chromosomal variants reported here. How did new regular chromosome numbers become established in the newly founded populations? How is it possible that this establishment rate is so high?

New chromosomal variants display hybrid dysfunction in *Carex* when crossed with other chromosome variants only if they differ in several chromosomal rearrangements (Whitkus, 1988; Escudero *et al.*, 2016). Whereas a single chromosome rearrangement is not underdominant in holocentrics, the accumulation of rearrangements is strongly underdominant (Whitkus, 1988; Escudero *et al.*, 2016), which may facilitate the establishment of new chromosomal variants in organisms with holocentric chromosomes (Lucek *et al.*, 2022). In the context of a hybrid dysfunction, drift (and also inbreeding and geographical isolation) has been suggested as one of the main causes leading to successful establishment of a new chromosomal variant (Rieseberg, 2001). Our genetic data reveal a drastic decrease in genetic diversity in the Huelva population (GUI) and an even more drastic decrease in the Seville populations (AZN1 and AZN2). This evidence suggests that genetic bottlenecks during the west-to-east population colonization process were a contributing factor to rapidly fix new stable karyotypes. Our data also suggest that the colonization of new populations was mediated by one or few individuals followed by selfing, which may have favoured the establishment of chromosomal variants in the new populations (as the single or few colonizing individuals already carried the chromosomal rearrangements).

The geographical distribution of chromosome numbers within the AZN1 population (Fig. 2) further supports the importance of drift as well as inbreeding (levels of homozygosity were extremely high) and geographical isolation (the 25 genotyped seeds were identical to the parental individuals) in the establishment of new chromosomal variants. First, the genetic data (SSR) suggest that all individuals in this population are genetically identical (Table 2), which indicates that this population was probably founded by only one or very few homozygotic individuals and that genetic drift has fixed a unique genotype in the whole population (Table 2). Second, despite this total absence of genetic diversity in the populations, we find a high diversity of chromosome numbers, which suggests that chromosome mutation in holocentrics may be faster (and precede) than SSR mutations in sedges. In addition, we found a dominant regular chromosome number 2*n* = 70 (and two additional very infrequent regular chromosome numbers 2*n* = 72 and 2*n* = 68, the latter is a new regular chromosome number found only in this population). This new regular chromosome 2*n* = 68 is highly spatially clustered (Fig. 2) with other individuals with irregular 2*n* = 68* (three individuals) and 2*n* = 70* (one individual), which also suggests that the origin of these infrequent chromosome numbers and configurations in the population may be linked. This process of rapid chromosome diversification within populations and foundation of new populations by one or a few individuals may explain the high chromosomal diversity that is typically found in many sedge species (Luceño and Castroviejo, 1991; Hipp *et al.*, 2010; Escudero *et al.*, 2013*a*, *b*).

## CONCLUSIONS

Chromosomal fissions and fusions are very important in the evolution of holocentric lineages, including sedges (Lucek *et al.*, 2022). In fact, there is a strong inverse association between mean chromosome size and number in sedges (Burchardt *et al.*, 2020; Elliot *et al.*, 2022). The evolution of fission and fusion in sedges has deep ecological consequences. For example, species in this lineage with smaller chromosomes have larger geographical distributions (Elliot *et al.*, 2022), and there is a relationship between chromosome number and climate regime (Escudero *et al.*, 2012, 2013*a*).

In the genus *Carex*, the hypothesis of recombination suppression in chromosomal speciation has not yet been tested. Nevertheless, changes in recombination patterns in holocentric taxa, alone or in combination with hybrid dysfunction, may be crucial for chromosomal speciation (Lucek *et al.*, 2022). Several experimental studies support the hybrid dysfunction model of chromosomal speciation in *Carex* (Whitkus, 1988; Escudero *et al.*, 2016). Drift and also inbreeding and geographical isolation are very important in hybrid dysfunction models of chromosomal speciation (Rieseberg, 2001). Our study adds additional experimental support to hybrid dysfunction models of chromosomal speciation in sedges as it illustrates with experimental data how new chromosomal variants are more easily established in the process of genetic bottlenecks increasing genetic drift, inbreeding and geographical isolation.

## SUPPLEMENTARY DATA

Supplementary data are available online at https://academic.oup.com/aob and consist of the following.

**Figure S1.** Location of all individuals during 2014 in the population AZN1. **Figure S2.** Neighbour-joining tree based on Bruvo’s distances, with population coding as in Table 1. **Figure S3.** Neighbour-joining tree based on Nei’s distances. Bootstrap support > 50 is indicated above branches. **Figure S4.** Delta*K* from STRUCTURE for each number of clusters from *K* = 2 to *K* = 10. **Figure S5.** Maximum clade credibility tree from BEASTvntr analyses. The *x*-axes indicate time in years. **Table S1.** Locus name, allele number, Simpson’s index of diversity, expected heterozygosity and evenness for each of the 34 analysed SSR loci.

mcad087_suppl_Supplementary_Material
