# Supplementary material for: Founder events and subsequent genetic bottlenecks underlie karyotype evolution in the Ibero-North African endemic Carex helodes
Source: Ann Bot. 2023 Jul 3;133(5-6):871–82. doi: 10.1093/aob/mcad087 (PMC11082475; doi:10.1093/aob/mcad087)
Supplement: mcad087_suppl_Supplementary_Material [file mcad087_suppl_supplementary_material.zip › aob-23320-s07.docx]

**Supplementary tables**

Table S1. Locus name, allele number, Simpson’s index of diversity, Expected heterozygosity and evenness are indicated for each of the 34 analyzed SSR loci.

| Locus name | Allele number | 1-D | Hexp | Evenness |
| --- | --- | --- | --- | --- |
| FamC2B | 5 | 0.4875 | 0.4889 | 0.6056 |
| Fam587 | 8 | 0.7199 | 0.7219 | 0.7598 |
| VicYES | 6 | 0.6212 | 0.6230 | 0.7745 |
| VicFG7 | 7 | 0.6138 | 0.6155 | 0.6115 |
| NedWE1 | 8 | 0.5664 | 0.5680 | 0.5918 |
| Ned121 | 8 | 0.7225 | 0.7246 | 0.6826 |
| FamT2N | 5 | 0.5507 | 0.5522 | 0.6576 |
| FamXAF | 7 | 0.6430 | 0.6449 | 0.7284 |
| Vic147 | 9 | 0.4386 | 0.4399 | 0.4957 |
| NedODD | 9 | 0.7555 | 0.7577 | 0.7221 |
| NedMIW | 3 | 0.4686 | 0.4699 | 0.8208 |
| PetGVB | 5 | 0.4799 | 0.4813 | 0.6081 |
| FamPM5 | 7 | 0.5585 | 0.5600 | 0.7351 |
| FamQ9D | 3 | 0.6549 | 0.6567 | 0.9738 |
| Vic468 | 6 | 0.2703 | 0.2711 | 0.4723 |
| Ned7LF | 14 | 0.6152 | 0.6170 | 0.5242 |
| PetQWB | 3 | 0.5458 | 0.5474 | 0.7886 |
| PetQWV | 3 | 0.5419 | 0.5434 | 0.8573 |
| FamPUI | 5 | 0.5838 | 0.5854 | 0.7526 |
| Vic222 | 6 | 0.4766 | 0.4779 | 0.5809 |
| NedEZE | 4 | 0.6387 | 0.6405 | 0.8199 |
| PetSGU | 5 | 0.5805 | 0.5821 | 0.6763 |
| Pet8RA | 4 | 0.2256 | 0.2263 | 0.5168 |
| PetORP | 6 | 0.5986 | 0.6003 | 0.7671 |
| PetBI5 | 3 | 0.6260 | 0.6278 | 0.9270 |
| Ned130 | 5 | 0.6160 | 0.6177 | 0.7256 |
| Fam829 | 12 | 0.6256 | 0.6274 | 0.5682 |
| Fam677 | 4 | 0.4619 | 0.4632 | 0.6934 |
| Fam84P | 5 | 0.2945 | 0.2953 | 0.5552 |
| FamWMU | 4 | 0.4978 | 0.4992 | 0.8085 |
| FamL7Z | 4 | 0.6286 | 0.6304 | 0.7732 |
| PetUAX | 4 | 0.6666 | 0.6685 | 0.9801 |
| Fam932 | 3 | 0.0494 | 0.0495 | 0.3846 |
| Vic192 | 3 | 0.5814 | 0.5830 | 0.8849 |
| mean | 5.6765 | 0.5414 | 0.5429 | 0.7007 |
