# Supplementary material for: Founder events and subsequent genetic bottlenecks underlie karyotype evolution in the Ibero-North African endemic Carex helodes
Source: Ann Bot. 2023 Jul 3;133(5-6):871–82. doi: 10.1093/aob/mcad087 (PMC11082475; doi:10.1093/aob/mcad087)
Supplement: mcad087_suppl_Supplementary_Material [file mcad087_suppl_supplementary_material.zip › aob-23320-s01.docx]

Supplementary Figure captions

**Figure S1.** Location of all individuals during the 2014 in the populations AZN1. Individuals with unknown chromosome number are indicated in white. The most common regular chromosome number 2n = 70 is shown in blue. The regular chromosome numbers 2n = 68 and 2n = 72 are shown in yellow and purple, respectively. The irregular chromosome numbers of configurations 2n = 68*, 2n = 69 and 2n = 70* are shown in orange, red and pink, respectively. Note: We use the asterisk to indicate the presence of irregularities in paired diploid chromosome numbers.

**Figure S2.** Neighbor-joining tree based on Bruvo’s distances, with population coding as in Table 1. Bootstrap support > 50 is indicated above branches.

**Figure S3.** Neighbor-joining tree based on Nei’s distances Bootstrap support > 50 is indicated above branches.

**Figure S4.** DeltaK from STRUCTURE for each number of clusters from K = 2 to K = 10.

**Figure S5.** Maximum clade credibility tree from BEASTvntr analyses. X axes indicate time in years.
