# Supplementary figures and images for: Founder events and subsequent genetic bottlenecks underlie karyotype evolution in the Ibero-North African endemic Carex helodes
Source: Ann Bot. 2023 Jul 3;133(5-6):871–82. doi: 10.1093/aob/mcad087 (PMC11082475; doi:10.1093/aob/mcad087)

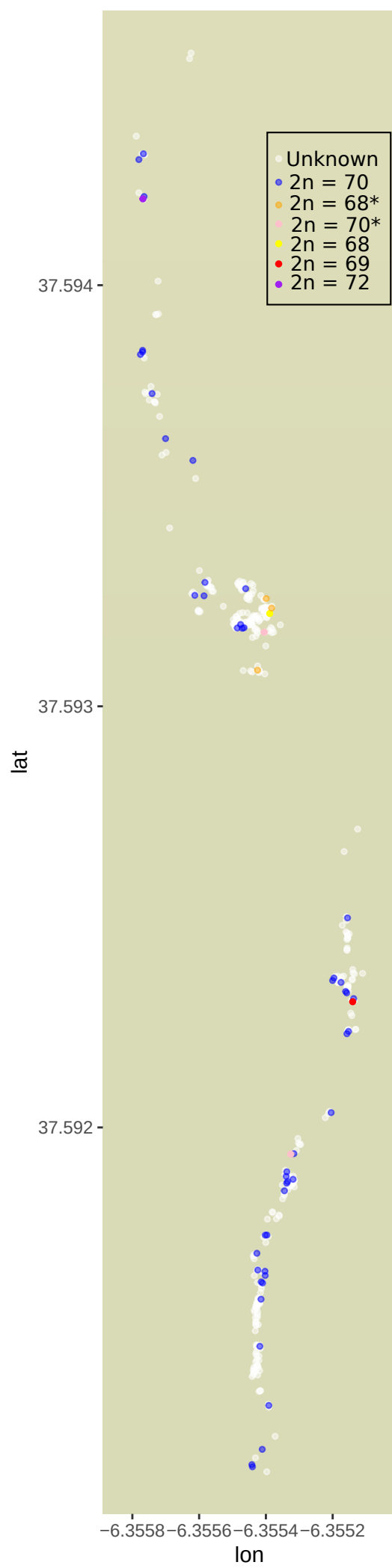

Supplement: mcad087_suppl_Supplementary_Material [file mcad087_suppl_supplementary_material.zip › aob-23320-s02.pdf]

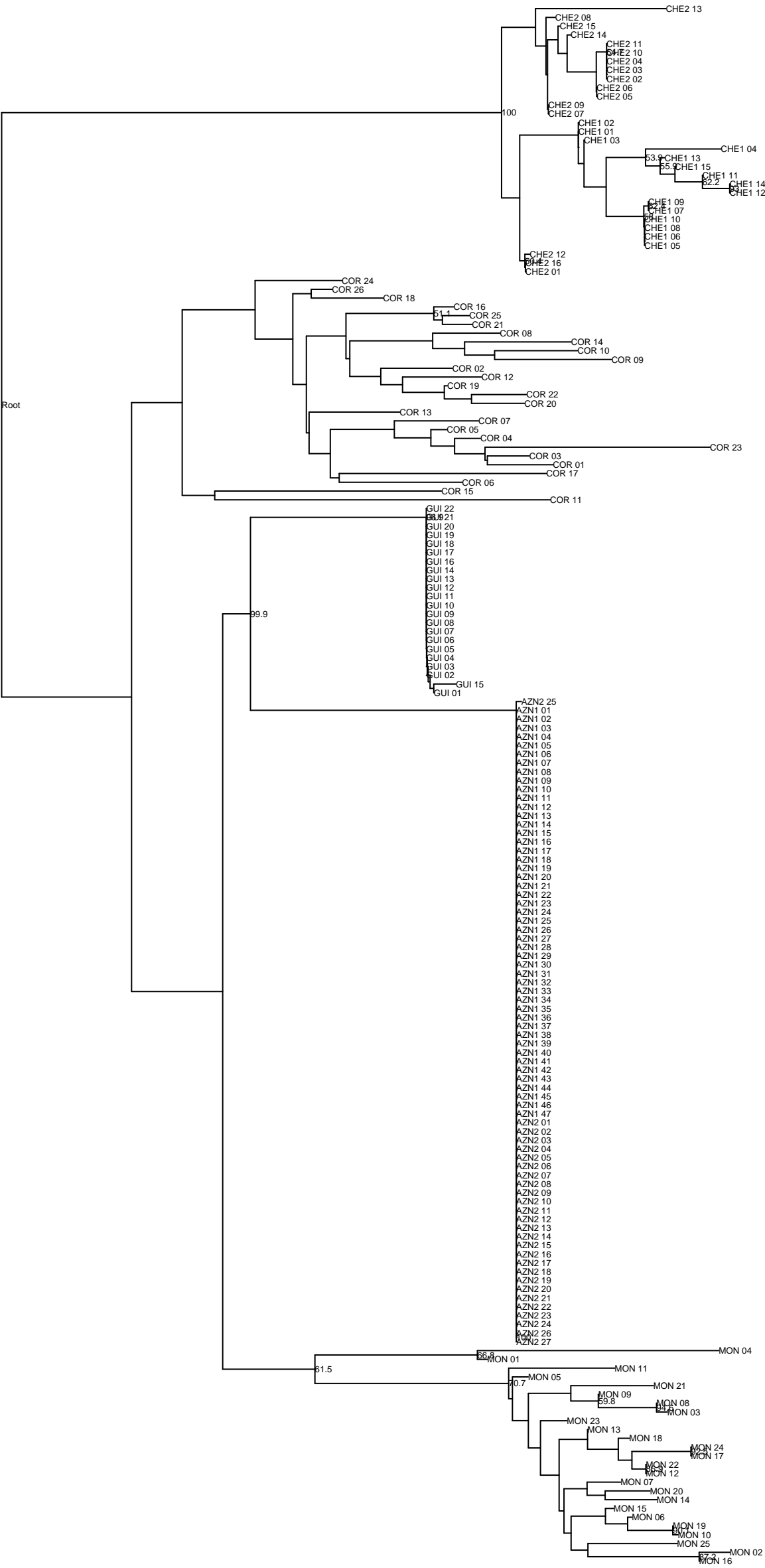

Supplement: mcad087_suppl_Supplementary_Material [file mcad087_suppl_supplementary_material.zip › aob-23320-s03.pdf]

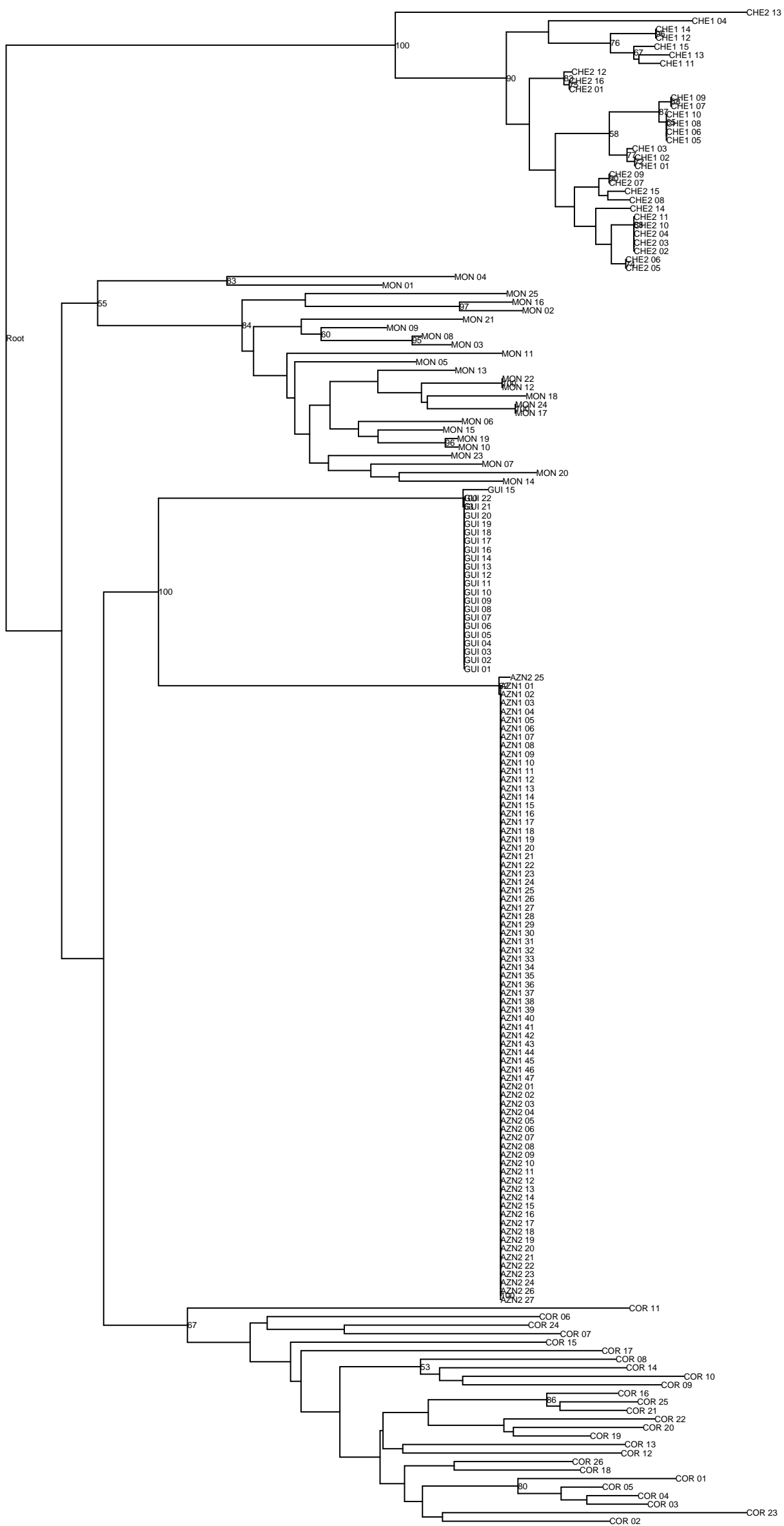

Supplement: mcad087_suppl_Supplementary_Material [file mcad087_suppl_supplementary_material.zip › aob-23320-s04.pdf]

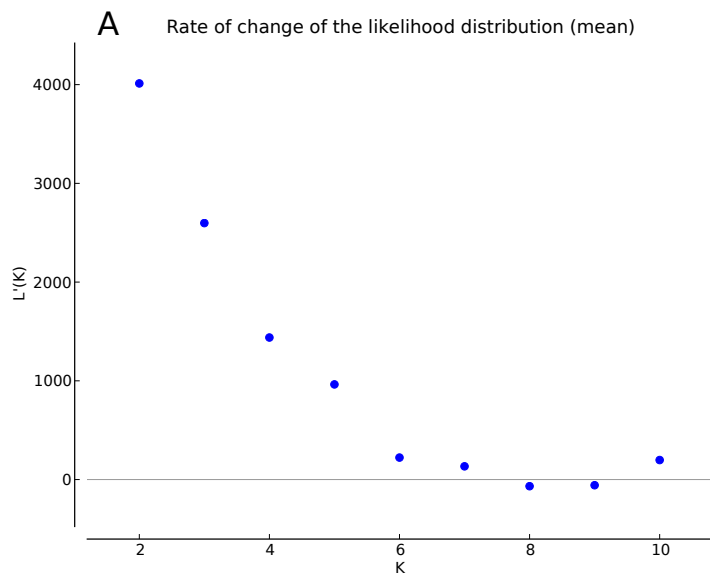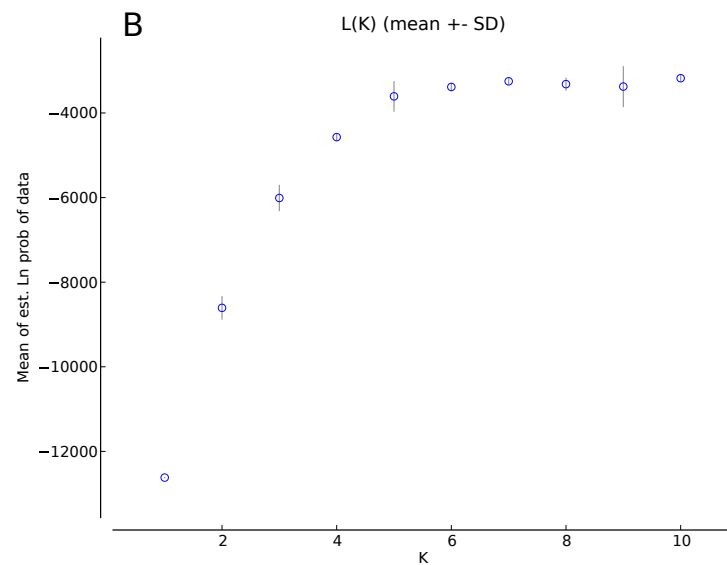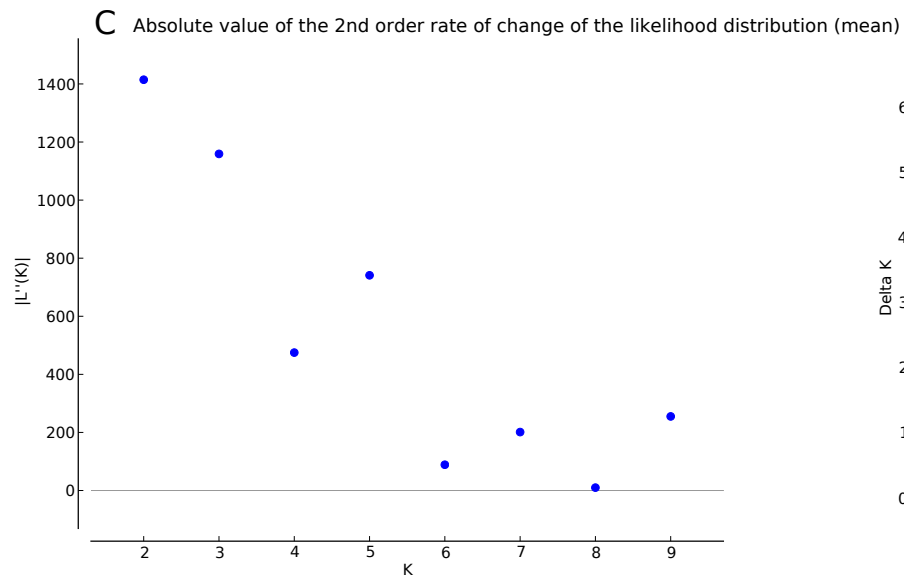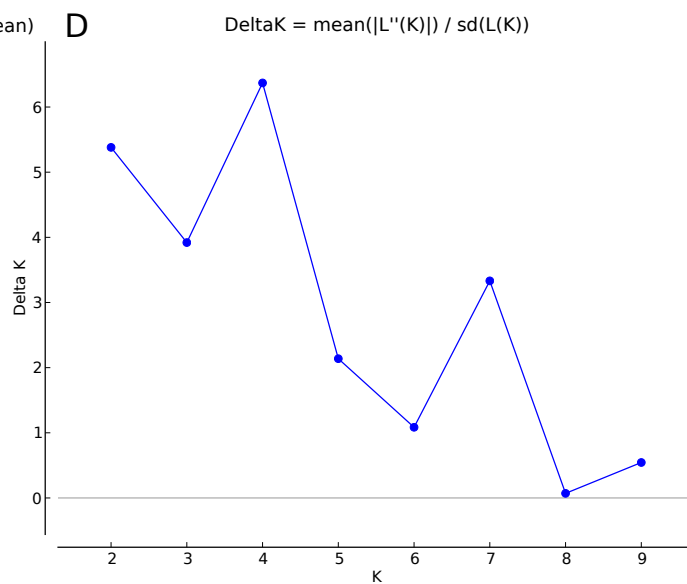

Supplement: mcad087_suppl_Supplementary_Material [file mcad087_suppl_supplementary_material.zip › aob-23320-s05.pdf]

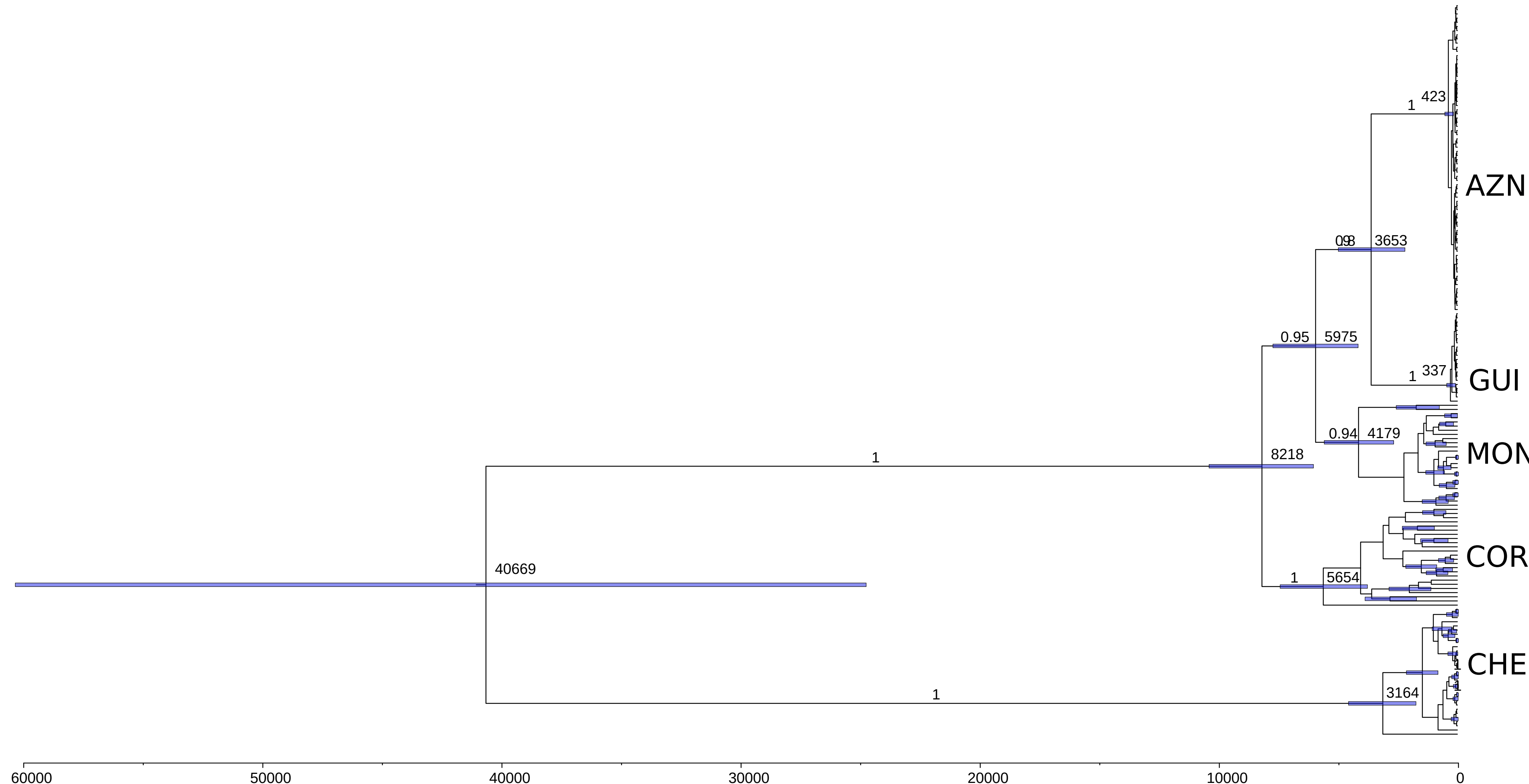

Supplement: mcad087_suppl_Supplementary_Material [file mcad087_suppl_supplementary_material.zip › aob-23320-s06.pdf]
